# Mechanism and disease association of E2-conjugating enzymes: lessons from UBE2T and UBE2L3

**DOI:** 10.1042/BCJ20160028

**Published:** 2016-10-11

**Authors:** Arno F. Alpi, Viduth Chaugule, Helen Walden

**Affiliations:** MRC Protein Phosphorylation and Ubiquitylation Unit, School of Life Sciences, University of Dundee, Dow Street, Dundee DD1 5EH, U.K.

**Keywords:** autoimmune disease, E2-conjugating enzyme, Fanconi anaemia, UBE2L3, UBE2T, ubiquitin signalling

## Abstract

Ubiquitin signalling is a fundamental eukaryotic regulatory system, controlling diverse cellular functions. A cascade of E1, E2, and E3 enzymes is required for assembly of distinct signals, whereas an array of deubiquitinases and ubiquitin-binding modules edit, remove, and translate the signals. In the centre of this cascade sits the E2-conjugating enzyme, relaying activated ubiquitin from the E1 activating enzyme to the substrate, usually via an E3 ubiquitin ligase. Many disease states are associated with dysfunction of ubiquitin signalling, with the E3s being a particular focus. However, recent evidence demonstrates that mutations or impairment of the E2s can lead to severe disease states, including chromosome instability syndromes, cancer predisposition, and immunological disorders. Given their relevance to diseases, E2s may represent an important class of therapeutic targets. In the present study, we review the current understanding of the mechanism of this important family of enzymes, and the role of selected E2s in disease.

## Introduction

**Figure 1.**
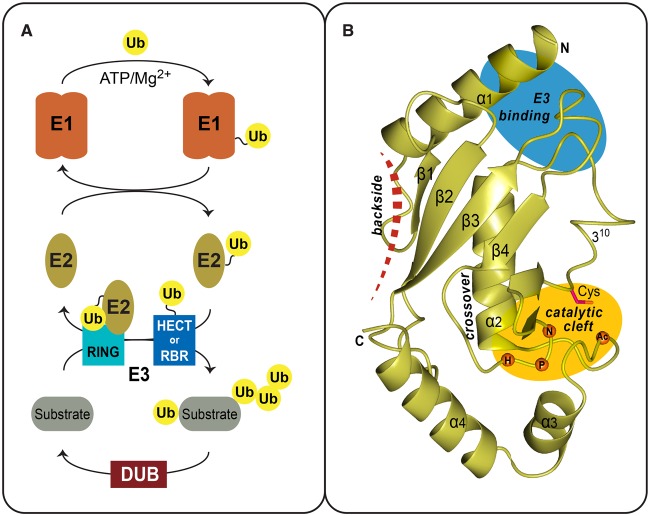
Ubiquitin pathway and the E2 fold. (**A**) Overview of the Ub pathway and the enzymes involved at each step: activation (E1), conjugation (E2), ligation (E3), and deubiquitination (DUB). The E1 mediates ubiquitin activation in an energy-consuming step. The ubiquitin thioester is then transferred onto a catalytic cysteine of the E2 enzyme. RING-type E3s form a non-covalent complex with the E2∼Ub thioester intermediate or, alternatively, ubiquitin is transferred to catalytic sites of HECT and RBR-type E3 ligases. The E3 enzymes ultimately catalyze ubiquitination of a substrate lysine. Ubiquitin signals can also be extended to form polyubiquitin chains. Finally, DUBs catalyze the removal of ubiquitin. (**B**) Ribbon diagram of UBE2D2 (PDB 2ESK) as a representative of the UBC fold conserved among ubiquitin E2s. Secondary structure elements and the termini of the domain are indicated. Also depicted is the location of the active-site cysteine within the catalytic cleft (yellow oval), the E3-binding region (blue oval), the cross-over helix that stabilizes ‘closed’ conformers of the E2∼Ub thioester intermediate and location of the E2 backside-binding surface (red dashed line).

Protein ubiquitination is a reversible post-translational modification that modulates a variety of homeostatic processes in eukaryotic cells. The modification typically involves the sequential action of activating enzymes (E1s), conjugating enzymes (E2s), and ligase enzymes (E3s), resulting in an isopeptide link between the C-terminus of ubiquitin (Ub) and a specific lysine on a target protein [1]. Briefly, E1s activate the C-terminus of ubiquitin in an ATP/Mg^2+^-dependent manner and load ubiquitin onto a conserved catalytic cysteine within E2s (denoted as E2∼Ub). Subsequently, hundreds of different E3s can then associate with one or more E2∼Ub to catalyze the ubiquitination of substrate targets (Figure 1A). The surface of ubiquitin itself presents several conserved functional patches, for example, the Ile44-hydrophobic patch (Leu8/Ile44/His68/Val70), which sustains crucial non-covalent interactions during ubiquitination as well as in signalling [2,3]. In addition, any of the seven surface lysines, or the N-terminus of ubiquitin, supports further building of ubiquitin chains that adopt distinct topologies [4]. Ubiquitin-binding domains are present within numerous proteins and can discriminate the different ubiquitin chain types, thereby facilitating signal cascades and protein networks [5]. Post-modification, enzymes called deubiquitinating enzymes (DUBs) can alter or erase the ubiquitin signals, thus modulating the event [6] (Figure 1A). Ubiquitin-like proteins (Ubls), such as SUMO (small ubiquitin-like modifier) and NEDD8 (neural precursor cell expressed developmentally down-regulated protein 8), also take part in post-translation modifications. These have a similar, but smaller, cohort of proteins that regulate the modification and have been reviewed elsewhere [7,8]. An intricate balance between E3 ligase and DUB activities regulates the specificity of the ubiquitin signal. Unsurprisingly, deregulation of these enzymes has been linked to several cancers and neurodegenerative diseases [9–12]. In addition to the large E3 and DUB classes, recent studies illustrate how E2s are more than just intermediaries of the ubiquitin pathway, but have multifaceted roles in human physiology and pathology. What follows is a review of our current mechanistic understanding of E2s, and the role of selected E2s in disease.

### Structure–function overview of the E2 fold

Humans have around 35 ubiquitin E2 family members, all sharing a core ubiquitin conjugation (UBC) domain that spans roughly 150 residues. Previously, E2s were classified by their UBC domain extensions; class I E2s have only the core domain, classes II and III have N- or C-terminal extensions respectively, and class IV are extended at both ends [13]. The extensions in the majority of classes II and III E2s are largely conserved, but lack secondary structure; they influence localization, E1/E3 interactions, or the ubiquitination event. Class IV enzymes, such as UBE2O and BIRC6, are significantly larger (>1200 residues) and are likely to be multidomain proteins. Currently, the *HUGO Gene Nomenclature Committee* has formally categorized mammalian E2s using the syntax UBE2X*n*, where X and *n* denote a letter and number, respectively (http://www.genenames.org/cgi-bin/genefamilies/set/102). UBC folds (Figure 1B) contain an N-terminal helix (α1), a four-stranded β-meander (β1–4), a short 3_10_-helix that leads into the central ‘cross-over’ helix (α2), and two C-terminal helices (α3 and 4) [14,15]. The E2 catalytic cleft is located in a shallow groove with the active-site cysteine preceding the 3_10_-helix. The catalytic cysteine is structurally supported by a tri-peptide His-Pro-Asn motif that is a conserved UBC fold feature [16,17]. Certain ubiquitin E2s vary slightly in the tri-peptide sequence, for example His-Pro-His in UBE2W, whereas others like the UBE2J and UBE2Q family completely lack the motif. Nevertheless, the variations do not abolish the ubiquitin conjugation activity in these E2s [18]. In contrast, the UBE2V family is devoid of both the catalytic cysteine and the His-Pro-Asn motif, and these proteins function as cofactors for the UBE2N enzyme. Also present in the catalytic cleft is a crucial, negatively charged, residue (typically Asp) in the α3–α4 loop that aligns the incoming substrate lysine towards the E2 catalytic cysteine [19]. Alternatively, in certain E2s, for example UBE2A, a neutral serine is present at the analogous position and phosphorylation of this residue can regulate the E2 enzyme function [20–23].

Ubiquitin can be activated by two distinct E1s: UBA1 and UBA6, with UBA1 capable of loading ubiquitin on the majority of E2s [24–26]. Structure–function studies of ubiquitin and Ubl E1s have uncovered details of the activation process and are reviewed elsewhere [27,28]. Interestingly, in mammalian cell lines, certain E2s (for example, the UBE2R family) are constitutively loaded with ubiquitin [25]. Structures of E2∼Ub thioester mimics reflect the dynamic conformations adopted by ubiquitin relative to the E2 [29–31]. In particular, the UBE2N∼Ub mimic resembles a compact ‘closed’ conformation more frequently than does a UBE2D3∼Ub mimic. The different conformers can be indicative of the ubiquitination potential of the E2. A closed E2∼Ub conformation is stabilized by interactions between the Ub Ile44-hydrophobic patch and conserved hydrophobic residues on the E2 cross-over helix. In UBE2S, UBE2R1, and UBE2G2, this closed conformation promotes efficient ubiquitin discharge, as well as E3-independent ubiquitin chain formation [32,33]. In addition, some E2s are capable of building linkage-specific polyubiquitin chains that involve additional interactions between the E2 and the acceptor ubiquitin surface surrounding the target lysine. In case of UBE2K [34,35], residues around the UBC catalytic cleft position the Lys48 surface of the acceptor ubiquitin, whereas C-terminal helices of UBE2S [33,36] orient the Lys-11 surface of the incoming ubiquitin. In contrast, the Lys63-specific UBE2N relies on an inactive E2 partner UBE2V1/2 to orientate the acceptor ubiquitin surface [37]. In the case of UBE2G2, homodimerization of E2∼Ub intermediate facilitates both donor and acceptor ubiquitin surfaces required for catalysis and results in assembly of Lys48-linked ubiquitin chains on E2 catalytic cysteine [38–40]. Other Lys48-specific E2s, including the UBE2G and UBE2R families [41–43], have insertions of variable lengths near the catalytic cleft. These facilitate linkage-specific products via different modes and have been reviewed elsewhere [44]. In the majority of cases, linkage-specific E2s extend previously monoubiquitinated substrates. This enables substrate polyubiquitination to be delineated into distinct events, that is ubiquitin chain initiation followed by elongation [45–47]. Initial ubiquitin conjugation, or lysine-specific monoubiquitination on substrates, almost always requires an E3. A contrasting example is the UBE2E family where disordered N-terminal extensions impede polyubiquitination events [48]. Remarkably, this E2 family mediates an E3-independent, site-specific monoubiquitination of the DNA methyltransferase SETDB1 and, consequently, stimulates the methyltransferase activity [49]. Furthermore, direct cross-talk between the DUB enzyme OTUB1 and several E2s, including the UBE2E family, is observed during DNA damage-associated ubiquitination [50]. Structural analyses reveal how ubiquitin from an E2∼Ub intermediate occupies the proximal ubiquitin-binding site of OTUB1, while free ubiquitin binds the distal site. The ternary E2∼Ub/OTUB1/Ub complex inhibits both ubiquitin discharge from the E2∼Ub intermediate and the DUB activity of OTUB1 [51,52].

Towards the N-terminus of the UBC fold are partially overlapping surfaces (α1, the β2–β3 loop, and the 3_10_-to-α2 loop) involved in interactions with both the E1 and E3 (Figure 1B). This overlap ensures that the E1–E2- and E2–E3-binding events are mutually exclusive during ubiquitin transfer, thus regulating the flow of the pathway [53,54]. Interactions of E2–E3 are usually weak and transient in nature, with dissociation constants in the high micromolar range. Conserved E2 residues that support the canonical E2–E3 interaction include polar residues in the N-terminal helix (typically Arg/Lys) and hydrophobic residues in the β2–β3 loop (typically Phe) and the 3_10_-to-α2 loop (typically Pro and Ala). Structures of the UBE2D family in complex with different E3s reveal how the E2 surface offers plasticity in the E2–E3 interaction [55–57]. Interestingly, E3 binding does not induce major conformational changes within the E2 UBC fold. Given that the E3-binding site is remote from the E2 catalytic cleft, the underlying mechanism forming a productive E2–E3 complex was not immediately clear. E3 ligases are broadly classified by how they catalyze the ubiquitination event. Briefly, really interesting new gene (RING)/U-box E3s (reviewed in ref. [58]) mediate direct transfer of ubiquitin from E2∼Ub to the target substrate, whereas the homologous to the E6-AP C-terminus (HECT) and RBR E3s (reviewed in refs [59,60]) form an additional ubiquitin thioester intermediate on a conserved catalytic cysteine (E3∼Ub) prior to substrate modification (Figure 1A). This distinction results in RING E3s being dependent on the partner E2 for regulating the ubiquitin product type, whereas HECT and RBR E3s can potentially overrule any linkage-specific bias. The different modes of ubiquitin transfer suggested that a productive E2∼Ub–E3 complex might differ depending on the type of E3 involved. Recent structural and biochemical analyses of the UBE2D family have revealed that HECT/RBR E3s do not require a closed E2∼Ub conformation to catalyze ubiquitination [61–64]. In contrast, RING/U-box E3s promote a closed E2∼Ub conformation, and mutations in the E2 cross-over helix that destabilize this result in the loss of ubiquitination [19,65,66]. Structures of an E2∼Ub mimic in complex with RING/U-box E3s also reveal critical contacts between E3s and ubiquitin that influence ubiquitination. First, a conserved E3 residue, typically Arg, makes connections with the Ile^36^ hydrophobic patch of ubiquitin as well as with the E2 main chain. These connections are essential for all RING/U-box-mediated ubiquitination; consequently, this residue has been denoted the allosteric ‘linchpin’. Second, the ubiquitin is further buttressed by contacts with homo-/hetero-dimeric partners of the RING domains, or by non-RING elements [19,65,66]. Taken together, these studies demonstrate how RING domain E3s, previously considered as mere molecular scaffolds, can actively influence the E2∼Ub intermediate for ubiquitination. Substrate binding and specificity, fundamentally an E3 attribute, can also involve direct E2–substrate interactions, as seen with the PCGF4 (polycomb group RING finger)/RING1B–UBE2D3 complex bound to the nucleosome target [67]. However, how the E2∼Ub/E3 modules interact with their non-ubiquitin-based target substrates is largely unexplored.

An interaction surface of increasing interest is the ‘E2 backside’ located on the β-meander, distal to the catalytic cleft (Figure 1B). In the UBE2D family, this surface supports non-covalent ubiquitin interactions that stimulate processivity of the E2 enzyme [68,69]. Several RING E3s also harbour ancillary E2 backside-binding regions that induce different allosteric effects, such as stabilization of the canonical RING–E2 interface, E2∼Ub charging, and processive ubiquitination [70–74]. Interestingly, in certain E2s, the backside interaction with E3s can regulate the type of ubiquitin signal generated on substrates [75,76]. Currently, functional roles for the E2 backside surface are observed in a quarter of all ubiquitin E2s and are predominantly coupled with RING E3s. Given the versatility of this surface, future work will undoubtedly reveal additional mechanisms that utilize the E2 backside to regulate ubiquitination.

Our current understanding of the mechanisms of E2 action is derived primarily from the extensively studied UBE2D family. However, there are 35 E2s, and an increasing number of them are associated with pathologies and diseases. Therefore, it is not surprising that there is a much greater diversity of action of E2 function than originally expected. Below, we discus UBE2T and UBE2L3 as specific examples of disease-associated E2s and describe our current understanding of their modes of action.

## UBE2T (FANCT), the E2 enzyme in the Fanconi anaemia pathway

### The Fanconi anaemia ubiquitin signalling module

An example of the importance of E2 function in disease is the role of UBE2T in the Fanconi anaemia syndrome (FA). The FA DNA repair pathway has become a paradigm for the physiological importance of ubiquitin signalling in coordination of DNA repair pathways and the maintenance of genome stability. In the late 1930s, the Swiss paediatrician Guido Fanconi reported a case of three brothers who all presented with developmental birth defects and eventually died of conditions reminiscent of aplastic anaemia [77]. The syndrome, now known as Fanconi anaemia, was recognized as a rare genetic disorder affecting bone marrow function and haematopoiesis. The combined efforts of many disciplines, ranging from paediatric haematology to biochemistry, determined the syndrome's molecular defects and uncovered the molecules and mechanism of a complex pathway that is dedicated to repairing DNA interstrand cross-links (ICLs). ICLs are induced after treatment with cross-linking agents such as mitomycin C (MMC) and cisplatin, as well as endogenously by metabolic by-products, most importantly reactive aldehydes. Pioneering work by Patel and colleagues provided compelling evidence of the interplay between aldehyde metabolism and the FA pathway [78–81]. Experiments carried out in mice revealed that increased aldehyde toxicity could trigger bone marrow failure, haematopoietic stem cell depletion, and the development of acute lymphoblastic leukaemia in FA-deficient mice. These defects indeed resemble the key phenotypes of FA patients, and it suggests that the FA pathway has an essential function in neutralizing aldehyde-induced damage in humans.

**Figure 2.**
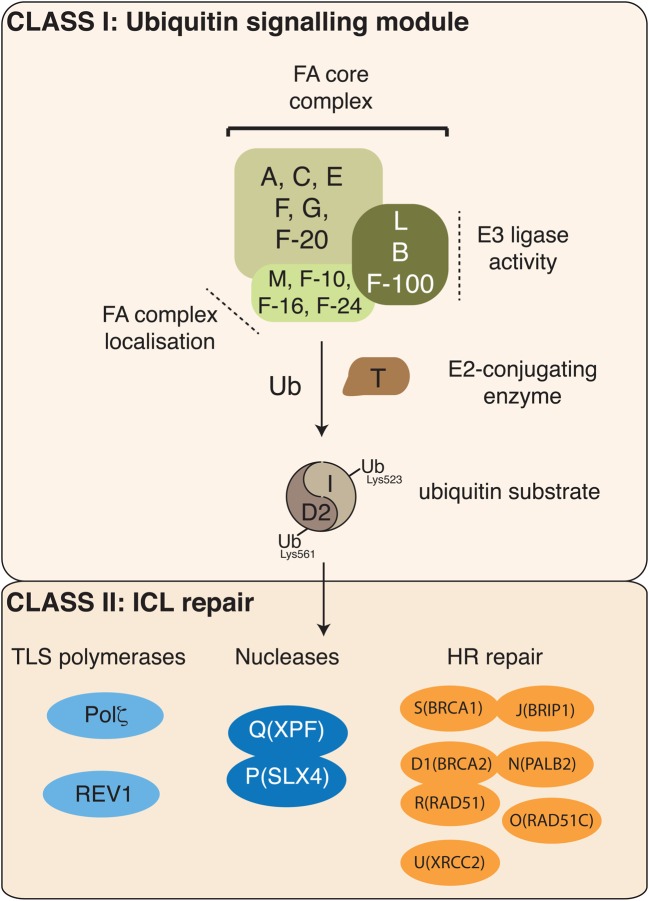
The Fanconi anaemia pathway. The FA pathway comprising 20 proteins is grouped into two classes: class I defines an upstream ubiquitin signalling module, and class II consists of downstream DNA repair proteins. The FA core complex is activated by stalled replication forks and co-operates with the E2-conjugating enzyme UBE2T (T) to monoubiquitinate the heterodimer FANCI-FANCD2 (I-D2). The RING E3 ligase FANCL (L) is the catalytic subunit, with associated FANCB (B) and FAAP-100 (F-100). Recruitment to stalled forks is mediated by FANCM (M), FAAP-10, -16, and -24. Monoubiquitinated I–D2 then promotes DNA repair by coordinating TLS (translesion synthesis) polymerase, nucleases, and HR-dependent pathways.

To date, 20 genes are associated with FA. These genes form two major classes based on their hierarchy and function within the FA pathway (Figure 2). The class I proteins FANCA, -B, -C, -D2, -E, -F, -G, -I, -L, -M, and UBE2T together with the FA-associated proteins (FAAP10, FAAP16, FAAP20, FAAP24, and FAAP100) comprise an ‘upstream’ ubiquitin signalling module. This group co-ordinates ‘downstream’ ICL repair by the class II proteins that include the nuclease scaffold protein SLX4 (FANCP), the structure­-specific nuclease XPF (FANCQ), and factors that are involved in homologous recombination repair: breast cancer gene 2 (BRCA2; FANCD1); PALB2 (FANCN); RAD51C (FANCO); RAD51 (FANCR); XRCC2 (FANCU); BRCA1 (FANCS); and FANCJ (BRIP1). The FA genes have been discussed in detail in several excellent reviews [82–85]; hence, we will focus on insights into the FA ubiquitin signalling module, in particular the E2-conjugating enzyme UBE2T.

The first evidence of an ubiquitin signalling module in FA came from the discovery of FANCD2 and its DNA damage-induced monoubiquitination [86]. Monoubiquitinated FANCD2 accumulates on chromatin, forming discrete foci at sites of DNA lesions, co-localizing with DNA repair factors. Any defects in FANCD2 monoubiquitination are as severe as FANCD2 loss-of-function mutations and result in hypersensitivity to ICL-inducing agents and elevated frequency of chromosomal abnormalities. Thus, it is thought that monoubiquitination is a key step in the signal transduction cascade of the FA pathway. Intriguingly, FANCD2 and the structurally related FA protein FANCI form a stable heterodimer FANCI–FANCD2 (I–D2), and FANCI is the second known substrate for targeted monoubiquitination in the FA pathway [87–89]. Site-specific monoubiquitination of the I–D2 complex on conserved lysine residues (FANCI^Lys523^ and FANCD2^Lys561^ in humans) is mediated by an E3 ubiquitin ligase, known as the FA core complex.

The FA core complex, first biochemically isolated by Meetei and colleagues, comprises 7 FA gene products FANCA, -B, -C, -E, -F, -G, -L, and the FA-associated proteins FAAP20 and FAAP100 [90–93]. In addition, the core complex associates with the DNA translocase FANCM and FANCM-associated factors FAAP10, -16, and -24 [94–98] and is thought to form a platform to recruit the FA core complex to damaged chromatin [96–98] (Figure 2). Within the FA complex, FANCL is the catalytic RING E3 subunit, and together with FANCB and FAAP100 constitute a minimal E3 module essential for I–D2 monoubiquitination in vertebrate cells [93,99–103].

The identification of the cognate E2-conjugating enzyme for FANCL was the last missing piece in the FA ubiquitin signalling module, as it is currently understood. Using a classical yeast two-hybrid approach, Machida et al. [104] found FANCL to be the predominant interaction partner of UBE2T. RNAi-mediated knockdown of UBE2T in human cells, or deletion of UBE2T in avian DT40 cells, abolished monoubiquitination of FANCD2, suggesting that UBE2T is likely the cognate E2 for FANCL in the catalysis of FANCD2 monoubiquitination [99,104]. This notion was further supported by *in vitro* reconstitution assays, showing that UBE2T and FANCL are necessary and sufficient to monoubiquitinate FANCD2 [100].

The UBE2T enzyme consists of a core UBC fold followed by a C-terminal extension (∼40 residues). The extension is largely unstructured, is poorly conserved and has negligible effect in FANCL-mediated FANCD2 monoubiquitination *in vitro* (Arno Alpi and Helen Walden, unpublished observations). A conserved lysine (Lys 91) near the UBE2T catalytic cleft is constitutively monoubiquitinated *in vivo* and has been proposed to negatively regulate the E2 [104]. Interestingly, UBE2T has unusually strong affinity for the FANCL RING domain (∼450 nM *K*_d_) [103]. Structural characterization of the FANCL RING–UBE2T pair reveals an interface area (∼700 Å^2^) that extends beyond the generic E3–E2 interface (typical area — 450 to 600 Å^2^), as well as several features that enable selective interaction for this E3–E2 pair [105]. In particular, an extended hydrophobic interface involves Tyr311 of FANCL anchored within an UBE2T pocket made up of Arg6, Arg9, and Asn103 side chains. This residue is highly variable in other RINGs and typically does not participate in the canonical RING–E2 interface. Furthermore, the β2–β3 loop of UBE2T bears a unique basic residue that forms a salt bridge with Glu340 of FANCL. This residue, Arg60 in UBE2T, is predominantly acidic (Asp/Glu) in other ubiquitin E2s and serves as a positive selector for the FANCL RING–UBE2T pairing.

Large-scale E2–E3 interaction studies propose interactions for UBE2T with at least 15 other E3s [18,106–108]. Notably, UBE2T is functional with a limited set of HECT E3s and primarily supports multi-monoubiquitination. However, the proposed UBE2T-binding RING E3s are poorly conserved with the FANCL RING domain; only five of them could possibly support a weak interaction, if any, with the β2–β3 loop in UBE2T. Experimental evidence for the E3s ARIH2 and RING finger 4 (RNF4) shows no significant ubiquitination products with UBE2T [109,110], while reports on a possible UBE2T–BRCA1 RING E3 pair are conflicting [111–113]. Nevertheless, FANCL was able to exclusively complex with UBE2T when presented with a group of different E2s and only the cognate E2–E3 pair resulted in the site-specific monoubiquitination of FANCD2 *in vitro* [105]. The specific and stable FANCL–UBE2T interaction offers insights into how a monoubiquitination event is regulated. As an overlapping UBE2T surface is required for both ubiquitin loading from the E1 and offloading via the E3, the low UBE2T off-rate could limit ubiquitin reloading, and thus curb incessant ubiquitination. However, we still do not completely understand how the FA core complex selects the precise lysine on I–D2 for ubiquitination. In addition, the allosteric ‘linchpin’ residue (typically Arg) required for all RING/U-box-mediated ubiquitination events is absent from FANCL (Ser363) [62]. Future structures of FANCL and/or the FA core complex bound to an UBE2T∼Ub intermediate and its substrate will ultimately reveal the determinants of this exquisitely specific ubiquitin signal.

### UBE2T is a *bona fide* FA gene

Almost a decade after the discovery of UBE2T in the FA pathway, three unrelated individuals (PNGS-252, PNGS-255, and 100166/1) diagnosed with FA have been described carrying biallelic mutations in the *UBE2T* gene locus [114–116]. Both PNGS-252 and PNGS-255 patients displayed FA-typical phenotypes including congenital malformations, haematological abnormalities, and high levels of chromosomal abnormalities that allowed a clear diagnosis. In contrast, 100166/1 was born with developmental abnormalities, but had normal bone marrow and blood counts that are atypical for FA. Despite this, he was eventually diagnosed with FA based on a diepoxybutane analysis revealing high frequencies of DEB(diepoxybutane)-induced chromosomal breakages in haematopoietic cells.

**Figure 3.**
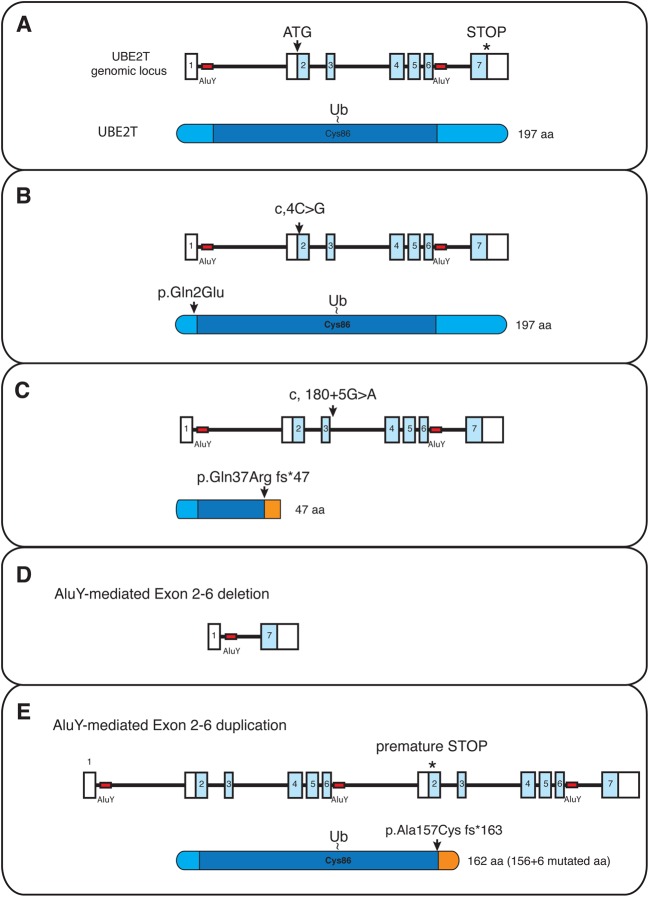
Schematic overview of identified *UBE2T* gene alterations in FA patients. (**A**) Genomic locus of UBE2T with intron/exon boundaries. Start codon in exon 2, stop codon in exon 7, and AluY repeats (red boxes) are indicated. UBE2T encodes a 197 aa protein with catalytic Cys86 in the UBC fold (dark blue). (**B**) Maternal missense mutation identified in patient PNGS-252 resulting in Gln2 to Glu2 amino acid substitution. (**C**) The c.180+5G>A splice donor site, identified in patient PNGS-255, initiating a frame shift and premature stop codon resulting in a truncated UBE2T protein. Genome alterations identified patient 100166/1 showing the paternal AluY-mediated deletion (**D**) and maternal AluY-mediated duplication (**E**). The maternal allele encodes an mRNA for a shorter UBE2T protein with a functional UBC fold.

Genome analysis for patient PNGS-252 revealed a maternal c.4C>G missense alteration resulting in the Gln2Glu amino acid substitution and a paternal 23 kb deletion across the *UBE2T* locus (Figure 3A,B) [114]. The Gln2 residue in helix1 of UBE2T is not an integral part of the UBE2T–FANCL interaction surface. However, the Gln2Glu mutation binds less well to FANCL and reduces FANCD2 monoubiquitination by UBE2T–FANCL *in vitro*. In agreement with this, fibroblasts derived from PNGS-252-expressing mutant UBE2T (Gln2Glu) are defective in ICL-induced FANCD2 monoubiquitination and display MMC hypersensitivity. Individual PNGS-255 had two allelic alterations; the c.4C>G mutation as found in PNGS-252 and, in addition, a c.180+5G>A splice donor site mutation [114]. This latter initiates a frame shift and premature stop codon resulting in a truncated UBE2T protein with a non-functional UBC domain (Figure 3B,C). Both alleles are considered to cause loss of function. The identification of the genome alterations in patient 100166/1 was challenging, as standard sequencing strategies including capillary Sanger and whole exome sequencing did not initially reveal any pathogenic mutations. Ultimately, two large AluY-mediated genome rearrangements were identified [115,116]. The patient inherited from the father a large intragenic deletion in UBE2T resulting from a recombination event between two AluY repeats, creating a new intron between exon 1 and 7. No functional protein is predicted to be expressed as the start codon resides in exon 2 (Figure 3D). Surprisingly, the same AluY repeats appeared to have initiated a recombination in the maternal allele that resulted in the large genomic duplication between these AluY repeats. The maternal allele encodes an mRNA for a truncated, but functional, UBE2T protein. However, the transcript can be detected only at low levels and is degraded by nonsense-mediated mRNA decay due to a premature stop codon (Figure 3E) [116]. Intriguingly, the maternal duplication of exons 2–6 seems to be reverted in the haematopoietic system of the patient and regenerated a wild-type allele, thus explaining that the normal blood counts observed in the patient were due to somatic mosaicism. Despite the fact that AluY repeats are apparently prone to rearrangements in the *UBE2T* genomic locus, further analysis of genomic DNA from normal individuals from Sicily (parents of patient 100166/1 are of Italian origin with ancestors from Sicily), Northern Italy, and Germany did, however, reveal extremely low frequencies of either AluY-mediated deletions or duplications of *UBE2T* exons 2–6 [116]. So far, all identified pathogenic alterations of UBE2T are likely loss-of-function alleles and the deficiency of UBE2T protein is associated with FA. Consequently, 10 years after the discovery of UBE2T protein function, *UBE2T* is now recognized as a *bona fide* FA gene and is alternatively named FANCT.

The specific and selective interaction of UBE2T with FANCL, as well as the typical FA-associated pathologies observed in UBE2T-deficient patients, suggests that UBE2T's primary function is dedicated to monoubiquitinate I–D2 in the FA DNA repair pathway. However, there is some evidence that UBE2T might be associated with ubiquitin signalling pathways other than FA. In contrast with FA core complex-deficient cells (including FANCL deficiency), UBE2T deletion in the avian cell line DT40 (a lymphoblastoid cell) caused hypersensitivity to UV light and cells are less efficient in removing genotoxic cyclobutane pyrimidine photoadducts [117]. Further somatic genetic analysis revealed a genetic link between UBE2T and the nucleotide excision repair gene XPA, suggesting an UBE2T-mediated signalling pathway required for efficient nucleotide excision repair of certain UV lesions. However, the putative E3 ligase and ubiquitin-targeted substrates remain to be identified and are aims for future investigations.

As a historical note, initial evidence suggested UBE2W as an alternative E2 that can associate and function with FANCL to monoubiquitinate FANCD2 [100,118]. However, recent discoveries addressing the genetics and distinct catalytic features of UBE2W argue against a direct function of the E2 in FA and DNA repair pathways. UBE2W contains non-canonical structural characteristics that catalyze monoubiquitin ligation to α-amino termini by favouring the recognition of substrates with intrinsically disordered N-termini rather than structured epitopes [119]. Thus, the observed monoubiquitination of FANCD2 mediated by UBE2W–FANCL *in vitro* is likely to be an N-terminal ligation and does not recapitulate the lysine site-specific monoubiquitination by UBE2T–FANCL. In agreement with this, deletion of UBE2W in human, mouse, and avian cells does not render them hypersensitive to any tested genotoxins, including ICL-inducing agents [101,120,121], nor is DNA damage-induced FANCD2 monoubiquitination affected [121]. UBE2W-deficient mice are susceptible to early postnatal lethality, show abnormal epidermal differentiation in the skin, and defective immune and male reproductive systems, but none of these pathologies are indicative for FA [120]. Cumulatively, the requirement of UBE2W in the DNA damage response is not essential, but might be part of a more complex regulatory network. Recent work reveals that UBE2W can functionally interact with the RING-type E3 RNF4 [110], which was shown to be essential for DNA double-strand break repair by both homologous recombination and non-homologous end-joining pathways [122,123]. By employing UBE2W, RNF4 is capable of promoting N-terminal monoubiquitination of SUMO chains *in vitro* [110]. However, genetic analysis reveals that simultaneous deletion of UBE2W in RNF4-deficient cells suppresses RNF4-associated DNA repair defects, suggesting that UBE2W and RNF4 function in different pathways [121]. To date, the biological function of UBE2W is far from being well understood and will be the subject for future investigations.

### UBE2T: potential oncogenic and tumour suppressor function in carcinogenesis

FA patients are highly susceptible to developing some form of malignancy throughout their lives with an observed-to-expected ratio of 48 over all cancers including leukaemia and solid tumours [124]. The majority of described leukaemias are acute myeloid leukaemia (AML), and the most common solid tumours are head and neck squamous cell carcinomas (HNSCC) [124]. FA patients deficient in genes of the homologous recombination repair pathway (FANCD1, FANCN, and FANCJ) are particularly prone to breast cancer [125–127], whereas FANCO mutations are associated with increased susceptibility to ovarian cancer [128,129]. So far, an accurate assessment of cancer predisposition linked with UBE2T loss of function is hampered by the limited number of affected patients. Patient PNGS-252 developed severe pancytopenia at age 13, successfully received bone marrow transplantation (BMT), and remains alive in haematological remission since then. Patient PNGS-255 developed AML at age 8. Recovery from a BMT from an HLA-mismatched-related donor was very poor and he died of a severe viral infection soon after [114].

**Figure 4.**
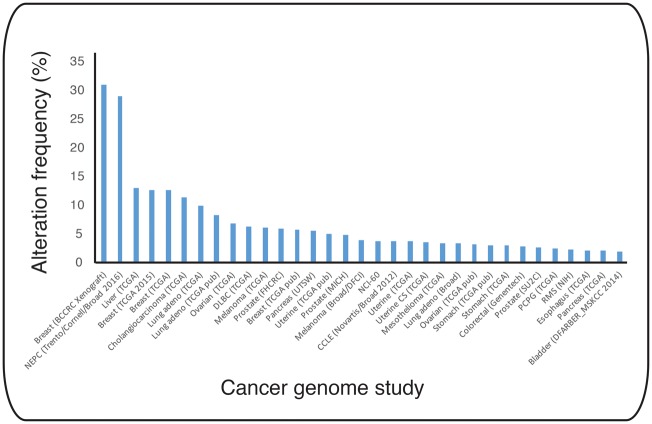
UBE2T amplifications described in human cancer studies. Graphic summary of frequencies of UBE2T alterations in cancers, mainly amplifications based on expression profile data from a panel of cancer cell lines deposited at cBioportal (http://www.cbioportal.org). Frequency of alteration is plotted against data from individual cancer genomic studies. TCGA (The cancer genome atlas), MSKCC (Memorial Sloan-Kettering Cancer Center).

Data mining in large-scale genome analysis and expression profile data from a panel of several hundred cancer cell lines deposited at cBioportal (http://www.cbioportal.org/) reveals that many cancers have alterations in UBE2T, in particular high frequencies of amplifications (Figure 4) [130]. Notably, UBE2T locates at 1q32.1, and the gain of 1q is frequently observed in a variety of cancers [131]. Several recent studies investigated UBE2T amplifications and UBE2T as a potential oncogene in different cancers. UBE2T is transcriptionally up-regulated with increased protein levels observed in breast cancers of different histological subtypes, including papillotubular, solid tubular, and scirrhous carcinoma [113]. The potential oncogenic function of elevated UBE2T is likely to be polyubiquitination and subsequent proteasomal degradation of the E3 RING ligase BRCA1 — a primary tumour suppressor associated with mammary carcinogenesis. In contrast, down-regulation of UBE2T by RNAi stabilized BRCA1 and blocked cell growth in breast cancer cells. *In vivo*, it is unclear whether UBE2T forms a direct, productive E2–E3 pair with BRCA1 or indirectly has an impact on the ubiquitin signal by associating with an, as yet unidentified, E3 ligase.

Elevated expression of UBE2T is also associated with lung, gastric, prostate, and nasopharyngeal cancer [132–135]. High UBE2T expression in gastric cancer tissue microarrays correlates with poor prognosis and was proposed as a potential prognostic marker in gastric cancer progression. Ectopic expression of UBE2T in gastric cancer cells promotes cell proliferation and induces epithelial–mesenchymal transition (EMT) by inhibiting the cell-to-cell adhesion factor E-cadherin, but elevating EMT-related factors N-cadherin, P-cadherin, and vimentin levels [135]. Moreover, similar studies of prostate cancer showed that increased UBE2T expression promoted proliferation and was sufficient to induce EMT of prostate cancer cells, as well as enhanced tumour growth in prostate cancer xenograft mouse models and prostate cancer metastasis [133]. Finally, it was demonstrated that UBE2T is highly expressed in nasopharyngeal carcinomas [134]. As in the aforementioned studies, UBE2T overexpression promotes growth of nasopharyngeal carcinoma xenografts and metastasis. Interestingly, elevated UBE2T expression in nasopharyngeal carcinomas activates the AKT/GSK3β/β-catenin pathway, and inhibition of the pathway with the AKT inhibitor MK-2206 2HCL blocks the pro-metastatic effect of UBE2T in nasopharyngeal cancers cells [134]. In summary, a common picture emerges from these cancer studies, suggesting that increased UBE2T protein levels and presumed increase in UBE2T activity promote cell transformation and cancer growth. UBE2T is less abundant in cells compared with other E2s, and its expression is tightly controlled [136]. Hence, non-physiological high UBE2T levels may trigger ubiquitination of signal transduction modulators, such as the AKT/GSK3β/β-catenin pathway and EMT factors, thereby either altering their activity or their cellular turnover by ubiquitin-target proteasomal degradation. Indeed, β-catenin and vimentin homeostasis is regulated by protein turnover. Given that UBE2T has an essential role in the FA DNA repair pathway, increased UBE2T may also trigger genome instability and subsequently cell transformation. Overall, UBE2T is likely to exhibit diverse roles that are context dependent in different cancer types. Efforts to identify UBE2T-specific substrates should be the ultimate goal in elucidating and dissecting the molecular mechanism of UBE2T in carcinogenesis.

## Disease association of UBE2L3 (UBCH7)

### UBE2L3 (UBCH7) in autoimmune disease

In recent years, several genome-wide association studies (GWAS) identified polymorphisms in the genomic locus of *UBE2L3* that are associated with multiple autoimmune diseases. Haplotype analysis shows that the *UBE2L3* locus has a simple structure encompassing one linkage disequilibrium block covering the majority of genetic variations and extending the full length of the gene locus (summarised in ref. [137]). Several single-nucleotide polymorphisms (SNPs) describe risk alleles associated with systemic lupus erythematosus [138–140], inflammatory bowel disease [141], Crohn's disease [142], rheumatoid arthritis [143], celiac disease [144], psoriasis [145], diffuse cutaneous systemic sclerosis [146], and juvenile idiopathic arthritis [147]. Notably, the risk allele rs5754217 shows significant association with systemic lupus erythematosus and rheumatoid arthritis, suggesting that both autoimmune rheumatological diseases may have *UBE2L3* as a common susceptibility locus [148]. A recent GWAS found that the *UBE2L3* locus is associated with chronic hepatitis virus B infection in Han Chinese patients, suggesting that UBE2L3 is required to clear hepatitis virus B infections. By employing expression quantitative trait locus analysis, the majority of *UBE2L3*-associated SNPs that conferred disease risks correlated with higher expression of UBE2L3. It is tempting to speculate that increased UBE2L3 expression and elevated UBE2L3 activity may be causative of altered immune response pathways triggering autoimmune diseases.

The UBE2L3 enzyme consists of an UBC domain and retains several structural features of the generic UBC fold. However, the catalytic cleft bears a histidine (His119) analogous to the acidic residue in the α3–α4 loop (D117 in UBE2D1) that orients the incoming substrate lysine. Early biochemical and structural studies describe UBE2L3 as predominantly functioning with HECT E3s [149–151]. A landmark study that compared E3-independent E2∼Ub reactivity profiles discovered UBE2L3∼Ub to be sensitive exclusively to thiol (cysteine) acceptors, whereas UBE2D3∼Ub could offload ubiquitin to both free lysines and cysteines [152]. Furthermore, the intrinsic property of UBE2L3 was unaffected by the presence of RING E3s. These data define UBE2L3 as primarily co-operating with HECT or RBR E3 ligases (RING1-BRcat-Rcat E3 ligases), wherein the E2∼Ub intermediate offloads ubiquitin onto the E3 catalytic cysteine rather than a substrate lysine.

UBE2L3 surface profiling reveals hot-spot residues (Lys9 in α1, Phe63 in the β2–β3 loop, and Glu93, Lys96, and Lys100 in the 3_10_-to-α2 loop) that drive HECT E3 interactions and consequent ubiquitination events [18,153]. Interestingly, the same surface is exploited by the pathogenic Shigella effector kinase, OspG, for binding the UBE2L3∼Ub intermediate [154,155]. This interaction impedes productive E2–E3 interactions and downstream ubiquitin signals during innate immune responses while concomitantly enhancing OspG kinase activity that abets pathogenic infection. Recent structure–function studies suggest that HECT/RBR E3s do not require a closed E2∼Ub conformation to promote ubiquitination [61–63]. In the case of UBE2L3, the RBR region of HHARI (human homologue of Ariadne) binds tightly with the E2 (roughly 200 nM *K*_d_) via an endothermic process [156]. Similar analysis with an activated Parkin E3 species reveals a strong binding with only the UBE2L3∼Ub intermediate (roughly 900 nM *K*_d_) [157]. Furthermore, kinetic analysis of the interaction between UBE2L3 and the HECT E3 E6-AP (human papillomavirus E6-associated protein) reveals the presence of an, as yet unidentified, E2∼Ub docking site that is required for E3∼Ub formation, and is distinct from the canonical E3–UBE2L3 interface [158]. Taken together, we are as yet unable to mechanistically define how UBE2L3 undertakes E3∼Ub loading as well as inducing polyubiquitination.

**Figure 5.**
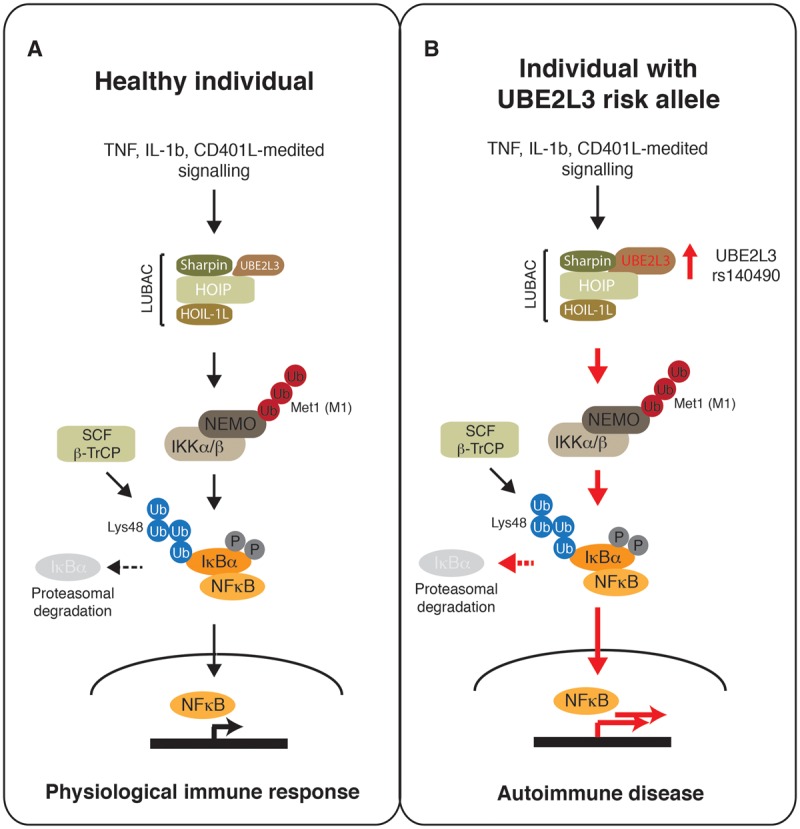
LUBAC and UBE2L3 regulate NF-κB response. (**A**) Cytokine/receptor-mediated activation of LUBAC/UBE2L3 triggers linear (Met1) polyubiquitination of NEMO required for IKK kinase activation. IKK phosphorylates the NF-κB sequestration protein IκBα that is then recognized by SCF/β-TrCP ligase for Lys48 polyubiquitination and subsequent degradation by the proteasome. Released NF-κB translocates to the nucleus to transactivate NF-κB response genes. (**B**) Model for hyperactivation (indicated by red arrows) of the NF-κB pathway in patients carrying UBE2L3 risk alleles associated with autoimmune diseases. UBE2L3 SNP rs140490 correlates with increased levels of UBE2L3 protein, causing enhanced LUBAC signalling, accelerated IκBα degradation, and hyperactive NF-κB response.

The intrinsic catalytic property of UBE2L3 restricts its functional alliance to a subset of E3s, namely the HECT-like E3 ligases and the specialized class of RBR E3s [18,152]. Nevertheless, deep proteomics of *in vivo* protein copy numbers indicate UBE2L3 as one of the most abundant E2s in mammalian cell lines [136]. Importantly, UBE2L3 can form productive E2–E3 pairs with the disease-associated E3 ligase LUBAC *in vitro* [159,160]. The 600 kDa RBR ligase complex LUBAC (linear ubiquitin chain assembly complex) associates with UBE2L3 and specifically forms linear (Met1-linked) ubiquitin chains *in vitro*. LUBAC is composed of HOIL-1L-interacting protein (HOIP), haem-oxidized IRP2 ubiquitin ligase-1 (HOIL-1L), and Sharpin (SHANK-associated RH domain interaction protein in postsynaptic density). Both HOIP and HOIL-1L proteins bear the multidomain signature seen in RBR E3s, but HOIP is thought to be the critical catalytic subunit of LUBAC. The E3 activity of HOIP is latent and is stimulated by heterodimer complex formation with either HOIL-1L or Sharpin. Several lines of genetic evidence suggest an important regulatory role of LUBAC and Met1-chain assembly in immune response signalling cascades (Figure 5A). LUBAC was shown to be critical for the efficient activation of NF-κB signalling, in part, by linear ubiquitination of NEMO, which is required to activate IKK kinase. IKK phosphorylates the NF-κB sequestration protein IκBα and triggers its degradation by the ubiquitin–proteasome system, releasing NF-κB during the initial phase of the NF-κB response. Importantly, recent work suggests that LUBAC participates in TNF receptor-mediated and genotoxin-induced apoptotic pathways by regulating death-promoting complexes in an inhibitory fashion [161–164]. Bi-allelic mutations of HOIL-1L were recently shown to underlie severe autoinflammation, immunodeficiency, and amylopectinosis [165] in human patients. HOIL-1L-deficient fibroblasts, and B-cells derived from patients, display impaired NF-κB activation in response to TLR agonists (TNF and IL-1β) and CD40L, respectively. In contrast, HOIL-1L-deficient monocytes are hyper-responsive to IL-1β, which probably accounts for the autoinflammatory phenotype in these patients. Recently, a patient with multiorgan autoinflammation and combined immunodeficiency was reported to bear a homozygous mutation in HOIP [166]. The cellular and clinical phenotypes largely overlap with those of HOIL-1L-deficient patients. In summary, there is overwhelming evidence that assembly and activity of LUBAC is required in a cell- and organ-dependent manner for various signalling pathways governing inflammation and immunity.

Given that UBE2L3 and LUBAC are both associated with autoimmune syndromes, an open question remained, until recently, as to whether UBE2L3 is a physiological E2 for LUBAC. Work by Lewis et al. [137] provided some insights into the underlying mechanism of UBE2L3 polymorphism and LUBAC function in systemic lupus erythematosus (Figure 5B). GWAS of systemic lupus erythematosus identified rs140490 as the most strongly associated SNP, which is located downstream of the promoter region of UBE2L3. Messenger RNA for UBE2L3 is increased in CD19^+^ B cells and CD4^+^ T cells isolated from peripheral blood of individuals with rs140490 genotype. However, rs140490 has increased UBE2L3 protein levels in only CD19^+^ B cells. Using NF-κB luciferase reporter cell lines, overexpression of UBE2L3 is not sufficient to increase basal NF-κB activation. However, co-expression of UBE2L3 with HOIP–HOIL-1L or HOIP–Sharpin triggers substantial up-regulation of NF-κB activity, particularly at the late phase of the biphasic NF-κB response. The effect is specific for UBE2L3; other E2s can form productive E2–E3 pairs with LUBAC *in vitro*, such as UBE2D1, UBE2D2, UBE2D3, or UBE2L6, but they do not affect NF-κB activation in the context of LUBAC subcomplexes. Down-regulation of UBE2L3, by siRNA, impairs phosphorylation and degradation of IκBα, similar to that observed in LUBAC-deficient cells [159,167–169]. Hence, UBE2L3 is likely to be the preferred E2 for LUBAC-mediated regulation of the NF-κB response. *Ex vivo* cell experiments demonstrate that the UBE2L3/rs140490 genotype increases basal and stimulated nuclear translocation of NF-κB in primary human B cells and monocytes (Figure 5B). Moreover, analysis of B-cell subsets from human blood samples reveals that the *UBE2L3* risk allele is specifically associated with increased levels of UBE2L3 in plasmablasts and plasma cells of systemic lupus erythematosus-affected patients, but not in healthy individuals. Taken together, these data suggest that increased abundance of UBE2L3 and an up-regulated NF-κB response may play a causative role in abnormal B-cell differentiation and proliferation in autoimmune diseases.

### Function of UBE2L3–HOIP in cancer

Hyperactivation of the NF-κB pathway is implicated in several blood cancers [170]. Active B cell-like (ABC) diffuse large B cell lymphomas (DLBCLs) are characterized by the constitutively activated NF-κB pathway, commonly due to gain-of-function mutations in the B-cell receptor and MYD88 pathways. Recently, screening biopsies of ABC-DLBCL have identified rare germline SNPs in the HOIP gene [171]. These SNPs increase the binding of HOIP to HOIL-1L resulting in an increased HOIP activity that contributes to NF-κB hyperactivation in ABC-DLBCL cells. It will be interesting to examine whether UBE2L3 is elevated in ABC-DLBCL cells and whether UBE2L3 is, as proposed by Lewis et al., a rate limiting factor for LUBAC activity. Hence, UBE2L3 might be a potential therapeutic target to treat SLE and B-cell lymphomas that are characterized by LUBAC hyperactivation and constitutive NF-κB signalling.

### Functional association of UBE2L3 with Parkinson's disease

Genotype and linkage analysis revealed that mutations in the *PARK2* gene, encoding Parkin RBR E3 ligase, are the most frequent cause of sporadic early- and late-onset Parkinson's disease (PD) cases [172,173]. In addition, *PARK2* was described as a putative tumour suppressor gene [174]. Parkin is thought to have an essential neuroprotective function by regulating mitophagy required for mitochondrial homeostasis and quality control. However, the identity of the cognate E2 that associates with Parkin in mitophagy and PD is less well defined. Parkin E3 ligase activity is auto-inhibited by the N-terminal UBL domain, which obscures the catalytic interaction between Parkin and the ubiquitin-loaded E2 [175–178]. Parkin's auto-inhibited state is relieved, and E3 ligase activated, by a dual PINK1 kinase-mediated phosphorylation event that involves phosphorylation of a crucial residue Ser65 on the UBL domain as well as binding of phosphor-Ser65 ubiquitin [179–183]. Subsequently, PINK1-activated Parkin allows the interaction with different ubiquitin-loaded E2s, including UBE2L3, to trigger and amplify ubiquitin signals at damaged mitochondria. Recent studies suggest a potential role of UBE2L3 in Parkin-dependent mitophagy [184,185]. Initial evidence came from *in situ* hybridization studies in rat brain tissues that indicate similar pattern and developmental changes in the expression of Parkin and UBE2L3 [186]. UBE2L3 is translocated to depolarized mitochondria dependent on active Parkin [184]. siRNA-mediated knockdown of UBE2L3 does not interfere with Parkin translocation, but impairs Parkin-dependent clearance of depolarized mitochondria [184,185]. Interestingly, only the simultaneous knockdown of UBE2L3, UBE2N, and UBE2D2/3 reduces ubiquitination of proteins of depolarized mitochondria [184]. Therefore, Parkin apparently employs different E2 enzymes to generate distinct ubiquitin chain signals that mediate efficient mitophagy. The apparent redundant functions of UBE2L3 with other E2s may explain the current lack of any genetic association of *UBE2L3* gene alterations with PD.

## Future perspectives

Our current understanding of E2 function includes substantial insights into the mechanisms common to all E2s. This field is now entering a phase of uncovering the differences between the enzymes that position each E2 in a given physiological context. This may also provide us with a framework to explain pathological functions of E2s associated with diseases. Indeed, the spectrum of pathologies and diseases associated with E2s is broad, ranging from immunological disorders and neurological syndromes (e.g. UBE2A [187]) to cancer, and undoubtedly, further involvement of E2s in other diseases will be discovered. The majority of E2s are linked with cancer of many different origins. The functional roles of several E2s in breast carcinogenesis have been described (reviewed in ref. [188]). For example, UBE2N is an emerging key player in various cancers, and the recently developed small compound inhibitor NSC697923 targeting UBE2N inhibits proliferation and survival of neuroblastoma and DLBCL cells. With the advent of enzymatic tools for trapping various states of E2 intermediates, there are now more opportunities to address the unique properties of E2s in different biological contexts and, importantly, to dissect disease pathways [189,190]. Certainly, this will pave the way to exploit E2s in therapeutic contexts, in particular by developing new strategies to target individual E2s or specific interactions of E2–E3 pairs.

## Abbreviations

AKT alias PKB, protein kinase B; AML, acute myeloid leukaemia; BARD1, BRCA1 associated RING domain protein 1; BMT, bone marrow transplantation; BRCA1, breast cancer gene 1; BRcat, benign required for catalysis; DEP, diepoxybutane; DLBCL, diffuse large B cell lymphomas; DUB, deubiquitylating enzyme; E6-AP, human papillomavirus E6- associated protein; EMT, epithelial–mesenchymal transition; eQTL, expression quantitative trait locus; FA, Fanconi anaemia; FAAP, FA-associated protein; GSK3, glycogen synthase kinase 3; GWSA, genome-wide association study; HECT, homologous to the E6-AP C-terminus; HHARI, human homolog of ariadne; HNSCC, head and neck squamous cell carcinomas; HOIL-1, haem-oxidized IRP2 ubiquitin ligase-1; HOIP, HOIL-1L interacting protein; ICL, inter-strand cross-link; IKK, IκB (inhibitor kappa B) kinase; LUBAC, linear ubiquitin chain assembly complex; MMC, mitomycin C; NEDD8, neural precursor cell expressed developmentally down-regulated protein 8; NEMO, nuclear factor κB essential modulator; PCGF, polycomb group RING finger; PCNA, proliferating cell nuclear antigen; PD, Parkinson’s disease; PTM, post-translational modification; RBR E3 ligases, RING1-BRcat-Rcat E3 ligases; Rcat, required for catalysis; RING, really interesting new gene; RNF, RING finger; SNP, single nucleotide polymorphism; SHARPIN, shank-associated RH domain-interacting protein; SLE, systemic lupus erythematosus; SUMO, small ubiquitin-like modifier; TLR, Toll-like receptor; TRIM, tripartite motif containing protein; Ub, ubiquitin; UBC, ubiquitin conjugation; UBD, ubiquitin-binding domain; UFD, ubiquitin-fold domain.

## Funding

This work was funded by Cancer Research UK (17739) (to H.W. and V.C.), the Medical Research Council (MC_UU_1206/12), a grant from the Scottish Government to The Scottish Institute for Cell Signalling (A.F.A.), and the EMBO Young Investigator Programme (H.W.).

## Competing Interests

The Authors declare that there are no competing interests associated with the manuscript.
